# The In Ovo Feeding Administration (*Gallus Gallus*)—An Emerging In Vivo Approach to Assess Bioactive Compounds with Potential Nutritional Benefits

**DOI:** 10.3390/nu10040418

**Published:** 2018-03-28

**Authors:** Tao Hou, Elad Tako

**Affiliations:** 1 College of Food Science and Technology, HuaZhong Agricultural University, Wuhan 430070, China; houtao@mail.hzau.edu.cn; 2 USDA-ARS, Robert W. Holley Center for Agriculture and Health, Cornell University, Ithaca, NY 14853, USA; Elad.Tako@ars.usda.gov

**Keywords:** intra-amniotic administration, in ovo feeding, prebiotics, mineral absorption, gut development, immune system

## Abstract

In recent years, the in ovo feeding in fertilized broiler (*Gallus gallus*) eggs approach was further developed and currently is widely applied in the evaluation process of the effects of functional foods (primarily plant origin compounds) on the functionality of the intestinal brush border membrane, as well as potential prebiotic properties and interactions with the intestinal microbial populations. This review collates the information of potential nutrients and their effects on the mineral absorption, gut development, brush border membrane functionality, and immune system. In addition, the advantages and limitations of the in ovo feeding method in the assessment of potential prebiotic effects of plant origin compounds is discussed.

## 1. Introduction

Functional foods, supplemented with bioactive substances (such as bioactive peptides, prebiotics, and polyphenols), provide health benefits and decrease the risk of chronic diseases [1,2,3,4]. Extensive research related to functional foods suggested numerous health benefits, including the decrease of cancer risk, improvement of heart health, enhancement of the immune system, improvement of gut health, diminution of blood pleasure, and decline of osteoporosis [5,6,7,8]. Among these functional foods are prebiotics, which are non-digestible food ingredients that beneficially affect the host by selectively stimulating the growth or activity of gut bacterial, and thereby exerting a health-promoting effect [9,10,11]. To date, prebiotics have been confirmed to have functional benefits such as the: (1) inhibition of acute gastroenteritis via the alteration of gut health and the immune system [1,12,13]; (2) reduction of cancer risk via the decrease of genotoxic enzyme production [14,15,16]; (3) promotion of uptake of minerals and release of bone-modulating factors [17,18,19]; and (4) the regulation of lipids [14,20]. 

**Figure 1:**
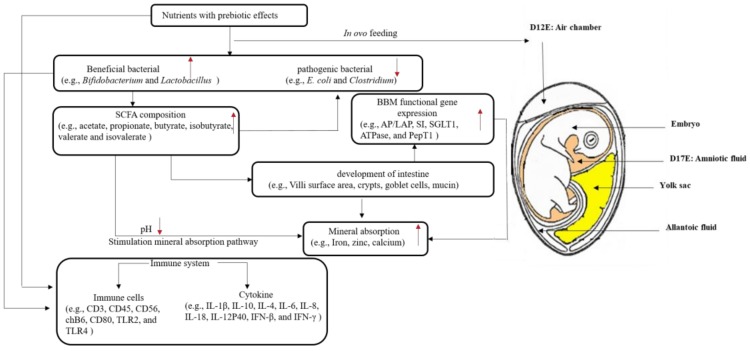
Schematic diagram depicting proposed mechanisms by which the in ovo feeding approach of nutrients with prebiotic properties may affect the *Gallus gallus* developing embryo. Processes described as follows: post in ovo administration, the gut bacterial populations are affected, mostly as the beneficial bacterial population’s increase (1). The increase of beneficial bacterial (such as *Bifidobacterium* and *Lactobacillus*) promote the production of short-chain fatty acids (SCFA) (2). The increased production of SCFA due to bacterial activity leads to a luminal pH reduction (3); moreover, intestinal morphology (such as villi height, crypts, goblet cells, and mucin) is affected (4), and the mineral absorption (iron, zinc, and calcium) is increased due to their pH reduction and their increased solubility (5). The morphological affects (increased villi surface area and goblet cell numbers) can potentially stimulate the intestinal functional genes expressions, primarily proteins that are required for intestinal mineral absorption. In addition, the in ovo prebiotic administration seemed to affect the immune system (6). Black arrow: the relationship between two factors; red arrow: increased or decreased levels. Injection target: the injection site is air chamber at Day 12 (D12;) the injection target is amniotic fluid at D17. BBM: Brush Border Membrane; AP: Aminopeptidase; LAP: leucine aminopeptidase; SI: Sucrase-isomaltase; SGLT1: Sodium glucose transporter 1; PepT1 peptide transporter 1; TLR: toll-like receptor; IL: interleukin; IFN: interferon; ATP: adenosine triphosphate.

Inulin, oligofructose, and galactooligosaccharides are the most intensively investigated prebiotics with regard to prebiotic effects [17,18,21,22]. Most of the studies on the effect of prebiotics have been performed in rats; the results showed that lactic acid bacteria increased in the intestine in the oligo-fructose treatment group after two weeks. However, in the long-term, any effect was lost in the rat animal model [23]. One study conducted in rats demonstrated that only inulin, alone, significantly (*p* < 0.05) increased bone mineral content (BMC) and density (BMD), and decreased the urinary excretion of collagen cross-links, which is a marker of bone resorption. However, oligo-fructose alone or oligo-fructose combined with inulin did not have an effect on BMC and BMD [24]. Silvi et al. (1999) used a human flora-associated rats model to look at the effects of resistant starch administration, and found that β-glucosidase increased, while β-glucuronidase and ammonia levels decreased [25]. In addition, a study on diabetic rats found that when xylooligosaccharides (XOS) replaced simple carbohydrates in the diet, the increase of serum cholesterol and triglyceride in diabetes were reduced, and liver triacylglycerols increased to a comparable level to that seen in healthy rats [26]. Moreover, human studies concluded no significant results [27,28], while others found that prebiotics stimulated calcium or magnesium absorption [29,30]. Tahiri et al. (2003) applied the metabolic balance and the stable isotope techniques in parallel, and found no significant effects (*p* > 0.05) of short-chain oligofructose on calcium absorption in postmenopausal women [27]. Ito et al. (1990) reported that feeding galactooligosaccharides to humans resulted in a decrease of nitroreductase, which is a metabolic activator carcinogenic substance. Meanwhile, the levels of indole and isovaleric acid as markers of putrefaction decreased in the galactooligosaccharide treatment groups [31]. Current research utilizes both animal and human models to evaluate the prebiotic potential effects of various nutrients; however, there are still inconsistencies in the results. In ovo exogenous nutrients administration was first applied in the 1980s for vaccination against Marek’s disease [32]. Over the years, further research on in ovo nutrients administration was conducted in order to potentially improve poultry production [33]. For example, numerous nutrients that have been applied for in ovo feeding, including amino acids [34], carbohydrates [35], and vitamins [36], are used to improve the quality of broiler chickens, specifically in the context of hatch weights, feed utilization, growth, and marketing size, all of which were observed to improve and increase post in ovo feeding [33]. Ohta and Kidd (2001) demonstrated that in ovo feeding site and time affect hatchability [34]. Figure 1 shows the various compartments that surround the poultry embryo (i.e., air chamber, albumen, yolk, allantoic fluid, and amniotic fluid). 

Previously, two time points during embryonic development were suggested and for the in ovo procedure. Both of these are on Day 12 (D12) of embryonic development, when the chorioallantoic membrane is fully developed and vascularized, and the embryo is surrounded by the amniotic fluid that remains in contact with the embryonic gastrointestinal tract, which allows the transport of substances from the air chamber into the intestine [37]. Villaluenga C.M. et al. (2004) demonstrated that the optimal time for the injection of a prebiotic is the 12th day of embryonic development. In comparison with injections on D1, 8, and 17, D12 injection resulted in a significantly (*p* < 0.05) increased relative abundance of intestinal *bifidobacteria* populations [38]. However, Uni and Ferket (2003) illustrated that in ovo feeding must be applied while the embryo consumes the amniotic fluid at 17–18 days of the embryonic development, just prior to the embryo’s oral consumption of the amniotic fluid, which occurs by Day 19 [39]. Salahi et al. (2011) provided evidence that the best in ovo injection time might be at 453 h of incubation [40]. It should be noted that the embryos are transferred from the setter to the hatching basket at D17–18, which should be an appropriate time to administer nutrients practically. Thus, the injection targeted egg compartment is amniotic fluid, on Day 17 of embryonic development. 

On either D12 or D17 (days of embryonic development), eggs were weighed and divided into relevant treatment groups. All of the treatment groups were assigned eggs of a similar weight frequency distribution. Next, each group was injected with a specified solution (1 mL per egg) with a 21-gauge needle into the air chamber or the amniotic fluid (days 12 or 17, respectively). The solution should maintain an osmolality value of ≤320 osmolality (OSM) in order to ensure that the embryo is not dehydrated. After all of the eggs were injected, the injection holes were sterilized and sealed with cellophane tape, and the eggs were placed in hatching baskets [35,39].

Currently, the in ovo feeding model is widely used as an in vivo method to assess the potential prebiotic effects, as shown in Table 1. Thus, the goal of this review is to focus on how nutrients present potential prebiotic effects by using the in ovo feeding method, particularly with reference to mineral absorption, gut microflora population, intestinal development, and short-chain fatty acids (SCFA) content. Hence, the potential of the in ovo feeding approach, as a technique for the evaluation of prebiotic effects is discussed.

## 2. In Ovo Administration and Mineral Absorption

### 2.1. Iron Status

Iron is a vital trace element for most life forms, and plays an important role in human health. Iron contributes to numerous biologic processes such as oxygen transport, DNA biosynthesis, and energy metabolism [59,60,61]. However, iron deficiency is the most common nutrient deficiency; it affects about two billion people worldwide [62]. The major causes of iron deficiency are the low iron content plant-based diets and low iron bioavailability [63]. Currently, a wealth of research aimed at exploring the effects of some substances (bioactive peptides and prebiotics) in the promotion of iron bioavailability and uptake is available [41,64]. For example, peptides from barley proteins were shown to increase iron uptake and ferritin levels in Caco-2 cells. Its SVNVPLY (Ser-Val-Asn-Val-Pro-Leu-Tyr) hexapeptide formed a chelate with Fe^2+^, which could increase Fe^2+^ uptake four-fold compared to FeSO_4_ [65]. β-lactoglobulin hydrolysate-iron complex could normalize hematocrit and hemoglobin, and improve serum iron levels in anemic rats [66]. Inulin and oligofructose have been shown to have benefits on the regeneration of hemoglobin mass, and increase intestinal iron absorption in anemic rats [67]. 

The intra-amniotic administration approach was used to evaluate the effect of natural occurring prebiotics in staple food crops on Fe bioavailability and absorption [54,68]. Previous research demonstrated that intra-amniotic administration and dietary inulin could increase ^58^Fe uptake, divalent metal transporter 1 (DMT1) gene expression, and liver ferritin amounts. In addition, the intestinal beneficial bacterial populations were also improved by inulin [54]. These results suggested that inulin improved iron status via changes in the bacterial population and the overall health of the intestine.

However, the study of intra-amniotic administration of wheat prebiotics demonstrated that there was no significant differences (*p* > 0.05) in the hatching Fe status and the intestinal expressions of DMT1, ferroportin, and duodenal cytochrome B (DyctB) between the treatment groups [52]. These results suggested that the iron status was not affected by the short-term exposure. Nevertheless, the study found an increase in the relative amounts of *bifidobacteria* and *lactobacilli* in the wheat prebiotics extract treatment group. This indicated that the iron bioavailability might be affected by wheat prebiotics in long-term studies via the increased production of short-chain fatty acids, due to bacterial activity, which lowers intestinal lumen pH, and hence increases iron solubility. 

Further, the intra-amniotic administration of raffinose and stachyose suggested that the prebiotic treatments up-regulated the relative expression of brush border membrane (BBM) functionality proteins, down-regulated the iron metabolism proteins, increased the relative abundance of beneficial probiotics and villi surface area, and decreased the pathogenic bacteria (*Clostridium* and *E. coli*) [41]. These results suggested that the intra-amniotic administration of raffinose and stachyose, compounds that are found in staple food crops such as chickpea and lentil [69,70], may improve iron status via bacterial activity. 

### 2.2. Zinc Status

Zinc is a required cofactor for the function of over 300 different enzymes, and participates in a wide variety of biochemical processes [71]. Zinc plays an important role in the regulation of genes involved in nucleic metabolism, cell signaling, and apoptosis [72,73]. Zinc deficiency is a major cause of stunting among children, who then run a risk of compromised cognitive development and physical capability [74]. 

Zinc cannot cross biological membranes by simple diffusion since it is a highly charged, hydrophilic ion [58]. Therefore, the uptake system in the intestine, such as the transport proteins, is paramount to zinc absorption [75,76]. Tako et al. (2005) used intra-amniotic zinc-methionine administration to evaluate the changes of the intestinal zinc exporter mRNA expression and small intestinal functionality. Authors found an approximately 200% mRNA increase of zinc transporter 1 (ZnT1) from 48 h post-ZnMet (zinc-methionine) injection compared to the control. Moreover, the gene expressions of the brush border enzymes and transporters showed increases of sucrase-isomaltase, leucine aminopeptidase, sodium–glucose cotransporter, and Na^+^/K^+^ATPase (Na^+^ and K^+^-stimulated adenosine triphosphatase) transporter (Na^+^/K^+^ATPase) from 48 h post-ZnMet injection. Meanwhile, the jejunal villus surface area increased significantly (*p* < 0.05) from the day of hatch (96 h post ZnMet injection). This study was the first introduction of the intra-amniotic administration approach in the evaluation of zinc digestion and BBM functionality [58].

Recently, the *Gallus gallus* was used to evaluate a proposed emerging physiological zinc status biomarker (the linoleic acid: dihomo-γ-linolenic acid ratio); this biomarker was assessed in the context of dietary zinc bioavailability in zinc biofortified staple food crops [77,78]. The broiler chicken model was used to explore the relationship between the dietary zinc deficiency and the red blood cell linoleic acid: dihomo-γ-linolenic acid ratio [78]. It was found that the linoleic acid: dihomo-γ-linolenic acid ratio significantly increased in the zinc dietary deficient group compared to that in the zinc adequate group (*p* < 0.001). Thus, the linoleic acid: dihomo-γ-linolenic acid ratio may be used as a biomarker of Zn status, specifically for the detection of marginal zinc deficiency status [79]. 

### 2.3. Calcium Status

Calcium (Ca^2+^), is an essential nutrient in the human body; as such, it participates in various biological pathways such as: intracellular metabolism, nerve conduction, blood muscle concentration, bone growth, and skeletal structural support [80,81]. Insufficient calcium uptake will cause bone resorption and the decrease of bone mass, which may lead to metabolic bone diseases, such as rickets in children and osteoporosis in the elderly [82]. Currently, some animal models, such as the calcium-deficient rat, are used for the in vivo assessment of calcium dietary bioavailability [8,17,19]. 

Several studies have shown positive effects of dietary prebiotics on calcium metabolism and bone composition [83,84,85]. The mechanisms by which prebiotics stimulate calcium absorption have been described and reviewed, and were suggested to be as follows [86,87,88,89]: (1) increased mineral solubility in the intestine due to the bacterial production of short-chain fatty acids; (2) enlargement of the absorption surface area by the promoting enterocytes proliferation; (3) stabilization of the intestinal flora and stimulation of gut beneficial prebiotics levels; (4) probiotic degradation of mineral-complexing phytic acid; and (5) increased expression of calcium-binding proteins. 

Additional research suggested that prebiotics improve bone heath by: (1) the release of bone-modulating factors; (2) the impact of modulating growth factors; and (3) the suppression of the bone resorption rate relative to the bone formation rate [90].

Recently, the intra-amniotic administration model was used to evaluate the effects of prebiotics and duck egg white peptides on the promotion of calcium uptake [42]. It was found that the prebiotics and peptides increased the relative abundance of beneficial probiotics, the intestinal villus surface area, and goblet cell diameters, as well as regulated the calcium-related gene expressions. This suggested that the chickpea prebiotic, lentil prebiotic, and duck egg white peptides are promising in improving Ca^2+^ status, and as was demonstrated by the in ovo feeding approach. Prebiotics from chickpea and lentil improve calcium bioavailability by promotion of gut beneficial prebiotics levels, the enlargement of gut villus surface area, and the improvement of BBM functionality. Duck egg white peptides promote calcium uptake through the reaction with calcium to act as calcium carriers and maintain gut health [42].

As shown in Figure 1, the administration of nutrients with potential prebiotics may increase the intestinal bacterial populations (such as *Bifidobacterium* and *Lactobacillus*); the fermentation activity of these populations leads to increased SCFA synthesis. The increased production of SCFA lowers the intestinal pH, and hence may increase mineral solubility [91]. 

## 3. In Ovo Administration and Small Intestinal Morphology

### 3.1. Intestinal Morphometric Parameters

The small intestine is highly specialized in the hydrolysis and absorption of nutrients, and constitutes the barrier between the host’s external and internal environment [37]. The intestinal villi play an essential role in the digestion and absorption processes of nutrients [37], as the villi increase the internal surface area, as well as the digestive and absorptive capacities of the brush border membrane (BBM) [92]. The intestinal epithelium that covers the villi is invaginated into the lamina propria, forming tubular glands called intestinal crypts [37]. The crypts are comprised of populations of continuously proliferating stem cells, which are responsible for the formation of various types of intestinal epithelial cells [93]. Amongst these cells are the enterocytes, which have a key role due to their nutrients’ absorptive ability from the intestinal lumen into blood vessels [93]. Deeper crypts lead to an increase in the secretion of digestive enzymes [94]. Thus, the surface area of the villi, the crypts’ depth, and the ratio between villi height and crypts’ depth are common indictors of intestinal developmental and functional status [94,95,96]. Hence, an increase of any of these morphometric parameters is expected to improve the digestive and absorptive capabilities of the BBM. 

In this context, the in ovo feeding of DiNovo (extract of *Laminaria* species of seaweed) significantly increased the width of duodenal villi and the depth of the crypts [37]. The villi surface area was also observed to increase post intra-amniotic administration of raffinose and stachyose [41,46], chickpea and lentil prebiotics [42], egg white peptides [42], symbiotic (inulin, *Enterococcus faecium*) [48], mannan oligosaccharides [56], carbohydrates (dextrin, maltose, sucrose) [57], and zinc-methionine [58]. However, in ovo injected probiotics (inulin, transgalactooligosaccharides, *Lactococcus lactis*) did not affect the villi heights, but rather changed the crypts’ depth [49]. The crypts’ depth increased after the injection of inulin combined with *Enterococcus faecium* [48] and mannan oligosaccharides [56], while the crypts’ depth was not affected by the injection of raffinose [46].

In the late embryonic and immediate post-hatch period, the small intestinal mucus-producing and secreting cells (goblet cell) begin to develop [39]. Since the intra-amniotic nutrients administration enhanced intestinal enterocytes proliferation, it may also affect the proliferation of goblet cells populations; this may further reflect on the intestinal digestive and absorptive capabilities. Pacifici et al. (2017) reported that the goblet cells’ diameters significantly increased post raffinose and stachyose administration [41]. A similar result was also observed post administration of mannan oligosaccharides [56]. In contrast, Calik et al. (2016) found that intra-amniotic symbiotic (0.5% inulin + 1 × 10^6^ *Enterococcus faecium*) administration had no effect on the goblet cell numbers, while dietary symbiotic treatments increased goblet cell numbers significantly [48]. In the case of mucin content, Smirnov et al. (2006) reported that carbohydrates injection led to an increased proportion of goblet cells containing acidic mucin compared with controls. On Day 19 of incubation (36 h after injection), the number of goblet cells containing acidic mucins was 50% greater than that in the controls [57]. More importantly, the mucin-secretion system was the first one to respond to the administration of the mannan oligosaccharides and the MUC2 (Mucin 2) gene expression increased three-fold compared to the control [56]. 

### 3.2. Microbial Populations

The intestinal microbial populations play an essential role in human and animal health [97,98,99,100]. It has been reported that the gut microbiome contains an estimated 3–8 million unique genes, which expands the genetic capacity of humans by >100-fold [101]. In recent years, it was demonstrated that the gut microbiome community participates in abundant bioactivities, such as the: (1) maturation and regulation of the immune system [102]; (2) digestion and release of essential nutrients [103]; (3) improvement of intestinal barrier function [90]; and the (4) potential inhibition of pathogenic bacteria [90]. 

*Gallus gallus* harbors a complex and dynamic gut microbiota [104], which is heavily influenced by host genetics, the environment, and diet [105]. There is considerable similarity at the phylum level between the gut microbiota of broilers (*Gallus gallus*) and humans, with Bacteroidetes, Firmicutes, Proteobacteria, and Actinobacteria representing the four dominant bacterial phyla in both [77,106]. Due to its rapid maturation and well-characterized phenotype, *Gallus gallus* has been used extensively as a model of human nutrition, especially as it pertains to assessing gut health and mineral absorption [58,77]. 

Recent studies suggested that cecal microbial populations are a useful indicator of gut health; this hypothesis was also confirmed in recent in ovo prebiotics administration studies [41,42,48,52,53,54]. For example, an early study showed that *Bifidobacterium* and *Lactobacillus* genera proportions were higher (*p* < 0.05) in intestinal contents of *Gallus gallus* after the intra-amniotic administration and dietary inulin treatment [54]. The increase of *Bifidobacterium* and *Lactobacillus* genera proportions were also observed in the intra-amniotic administration of wheat prebiotics [52], raffinose, stachyose [41], and chickpea and lentil extracts [42].

However, in the intra-amniotic administration of soy bean daidzein, there were no significant increases in *Bifidobacterium*, *Lactobacillus,* and *Clostridium* genera relative abundance in the 2.5 mg/mL daidzein treatment group. However, the relative *E. coli* abundance was significantly elevated [53]. The authors thought that *E. coli* might represent a candidate bacterial species involved in the biotransformation of daidzein to the bioactive metabolites, equol and *O*-desmethylangolensin [53]. Two prebiotics (DN, an extract of beta-glucans; and BI, transgalactooligosaccharides) both numerically increased the relative abundance of *Bifidobacterium* and *Lactobacillus* in chicken feces [43]. Similarly, the number of *Bifidobacterium* and *Lactobacillus* was consistently higher for both intra-amniotic administration and dietary symbiotic (inulin, *Enterococcus faecium*) treatments [48]. Moreover, the relative abundance of *Clostridium* significantly (*p* < 0.05) decreased in the presence of both concentrations of stachyose and raffinose compared to the controls, while the relative abundance of *E. coli* was not affected [41]. Interestingly, the relative abundance of *E. coli* and *Clostridium* significantly increased (*p* < 0.05) in the 18 MΩ H_2_O and Ca groups, and significantly decreased (*p* < 0.05) in peptide treatment groups compared to the non-injected group. The possible reason might be that prebiotics from chickpea and lentil and peptides from egg white could limit the presence of potentially pathogenic bacterial populations [42].

### 3.3. Short-Chain Fatty Acid Composition

The composition of SCFA in the intestine is significant to the mineral absorption of calcium, iron, zinc, and other micronutrients [90,92,107,108]. Previous research demonstrated that many prebiotics (i.e., soluble corn fiber, inulin, and agave fructans) increased the cecal content of SCFA, such as acetate, propionate, butyrate, isobutyrate, valerate, and isovalerate [4,90,109]. As the production of SCFA increases the intestinal lumen acidity, it also thus lowers the intestinal lumen pH and enhances minerals solubility (as Ca), which may lead to increased absorption [110,111]. 

The in ovo feeding model has been used to evaluate prebiotics and synbiotics (inulin with *Lactococcus lactis* subsp. *lactis* IBB SL1) on the cecal fermentation. The results showed that the propionate molar proportion was the highest in the groups treated with synbiotics, especially in the inulin with *Lactococcus lactis* subsp. *lactis* IBB SL1 group (Syn-1) (*p* < 0.01). In addition, the molar proportion of acetate was the lowest in the Syn-1 group (*p* < 0.05). However, the total cecal SCFA concentrations were similar in all of the groups, and the inulin group exhibited the lowest SCFA level. Other SCFAs, such as isobutyrate, isovalerate, valerate, isocaproate, and caproate, were low and not affected by the in ovo injections. Interestingly, the SCFA proportions varied over time in that study. The acetate molar proportion decreased, while the propionate and butyrate proportion increased [49]. Calik et al. (2016) used an approach that combined intra-amniotic with dietary administration to evaluate the effect of inulin with *Enterococcus faecium* on the SCFA composition [48]. Authors found that the butyrate concentration in the synbiotic group increased by 14.6% in comparison to the control group; however, this increase was not significant. In the dietary study, symbiotic (1.0% inulin + 2 × 10^9^ *Enterococcus faecium* cfu/kg feed) supplementation significantly increased the butyrate concentration at the end of the experiment [48]. Butyric acid has been shown to be the preferred energy source for enterocytes, and takes part in cellular differentiation and proliferation with the intestinal mucosa [112]. Additional research is needed in order to further investigate the efficacy and efficiency of the combined administration of prebiotics and synbiotics to increased SCFA synthesis, its potential effect on the intestinal probiotic populations, and in the context of intestinal functionality and development. 

### 3.4. Brush Border Membrane (BBM) Gene Expressions

**Figure 2:**
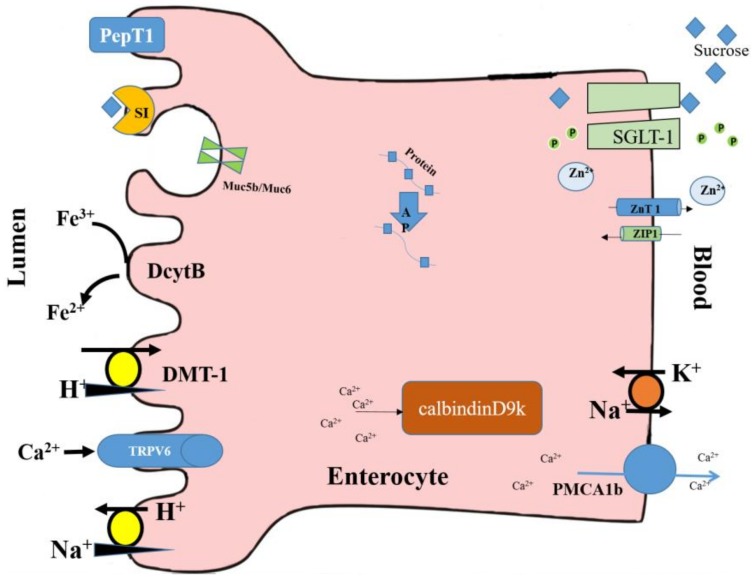
Schematic diagram of the discussed functional proteins located on the small intestinal enterocyte’s brush border and basolateral membranes. PepT1: peptide transporter 1; Dcyt B: duodenal cytochrome B; DMT-1: divalent metal transporter 1; AP: aminopeptidase; LAP: leucine aminopeptidase; SI: sucrase-isomaltase; SGLT1: sodium glucose transporter 1; TRPV6: transient receptor potential cation channel, subfamily V, member 6; PMCA1b: plasma membrane calcium ATPase 1b; calbindinD9k: calcium-binding protein.

BBM functional genes expressions are used as biomarkers of BBM digestive and absorptive capabilities and overall tissue functionality [77,113]. Aminopeptidase (AP) and leucine aminopeptidase (LAP) are enzymes that catalyze the cleavage of amino acids from the amino terminus (N-terminus) of proteins or peptides. Sucrase-isomaltase (SI) is a glucosidase enzyme that is located on the brush border of the small intestine. Sodium glucose transporter 1 (SGLT1) is a glucose transporter that is found in the intestinal mucosa (enterocytes) of the small intestine. ATPase is an enzyme that catalyzes the decomposition of ATP into ADP and a free phosphate ion. PepT1 (peptide transporter 1) is a solute carrier for oligopeptides; it functions in renal oligopeptide reabsorption, and in the intestines in a proton-dependent way. These functional proteins are all located on the enterocyte’s brush border and basal membranes, as shown in Figure 2.

One or more gene expressions from AP, SI, ATPase, and SGLT1 were significantly up-regulated by the intra-amniotic administration of chickpea and lentil prebiotics [42], stachyose and raffinose [41], daidzein [53], and zinc-methionine [58]. Cheled-Shoval et al. (2011) found that there was a five-fold increase in AP mRNA expression, and a two-fold increase in SI mRNA expression post mannan oligosaccharides in ovo treatment [56]. Chicken embryos have a limited ability to digest and absorb nutrients prior to hatching due to the low functional mRNA expression, such as AP, SI, ATPase, and SGLT1 in the small intestinal mucosa [39]. In the *Gallus gallus* model, immediate feeding post hatch is critical for the intestinal development [114]; therefore, nutrients supply via in ovo feeding enhances the intestinal development during embryonic development [33]. As shown in Figure 1, the up-regulation of the BBM functional genes expressions reflects the intestinal development and digestive capabilities. Thus, it also affects the potential increased absorption of nutrients as Fe, thus improving the poor Fe status of the late term embryo and Fe status post hatch. 

## 4. In Ovo Administration and the Immune System

Previous research indicated that the in ovo feeding approach improved early immune response [33]. Bhanja et al. (2010) reported that a higher expression of genes associated with humoral immunity, IL-6, and TNF-α, was observed after the treatment with lysine, threonine, or methionine and cystine [115]. Additionally, in ovo treatment of 10% glucose improved humoral immune response [116].

Schley et al. (2002) reported that the immunity system was modulated by prebiotics directly through the interaction with immune cell receptors, stimulation of endocytosis, phagocytosis, respiratory burst, and the production of numerous cytokines and chemokines [117]. Probiotics cross the intestinal barrier through intestinal epithelial cells, are processed and presented to the immune system, and modulate both the innate and adaptive responses [118]. As shown in Table 2 (section B), the expression of CD3, CD45, CD56, chB6, CD80, (toll-like receptor) TLR2, and TLR4 is frequently used as an indicator of immune response post prebiotic in ovo administration. In addition, some cytokines, such as IL-1β, IL-10, IL-4, IL-6, IL-8, IL-18, IL-12P40, IFN-β, and IFN-γ, are also used as indicators of immune status (Table 2, section C). CD3 is membrane protein that is expressed in T cells and used as a biomarker of T-cell activity [119]. CD56 is expressed on the surface of neurons, glia, skeletal muscle, and natural killer cells (NK), and is used as markers of NK cells with TLR2 and TLR4 [46]. chB6, which is also used as marker, is expressed in mature B cells [120]. CD80 is a costimulatory molecular marker that is expressed in T cells or B cells [50]. IL-1β and IL-10 are known as a pro-inflammatory cytokine and an anti-inflammatory cytokine, respectively. 

T helper-1 genes (IFN-β, IFN-γ, and IL-18), T helper-4 gene (IL-4), pro-inflammatory cytokine (IL-6 and IL-12P40), and a chemokine (IL-8) are all markers of the immune system. Berrocoso et al. (2016) reported that the expression levels of CD3 and chB6 in the small intestine of broilers was significantly (*p* < 0.05) up-regulated by raffinose administration [46]. Additionally, no significant difference was observed in the expression levels of CD56, TLR4, IL-1β, and IL-10 post raffinose injected broilers [46]. Madej and Bednarczyk (2016) studied the effect of in ovo feeding of prebiotics and synbiotics (inulin, transgalactooligosaccharides, *Lactococcus lactis subsp*. *lactis* IBB SL1 or *Lactococcus lactis subsp. cremoris* IBB SC1) on the composition of T cells and B cells in gut-associated lymphoid tissue. They found that the number of CD3-expressed cells was increased by some symbiotic; however, there was no significant difference on the population of CD3 or chB6-expressing cells in only prebiotics-treated birds [121]. TLR2 and TLR4 mRNA expression were significantly (*p* < 0.05) higher after the treatment with mannan oligosaccharides [56]. However, in ovo administration of inulin or inulin supplemented with *L lactis subsp lactis* 2955 on Day 12 of embryonic development resulted in a general down-regulation of immune-related genes in the spleen and cecal tonsils of broilers during the 35 days after hatching. The magnitude of that down-regulation increased with age, and was most likely caused by the stabilization of the gastrointestinal microbiota [50].

## 5. Conclusions and Future work

The evidence provided in this review demonstrate that in ovo feeding (primarily the intra-amniotic fluid administration) approach is a useful and time–cost effective in vivo method to evaluate the probiotic effects of nutrients. To date, research has shown that utilizing the in ovo feeding model of various plant origin prebiotics, peptides, isoflavones, carbohydrates, and synbiotics resulted in an in vivo indication of these compounds’ prebiotic effects (such as: mineral absorption, gut microflora population, intestinal development, short-chain fatty acid content, and immune system response). 

Future research via the utilizing the in ovo feeding model will be focused on the further identification of plant origin nutrients and bioactive compounds, which may improve intestinal overall health, and specifically the functionality of the digestive and absorptive surface, and beneficial bacterial populations. Current evidence indicates that the in ovo approach allows the investigation of a single nutrient or in combination of other ingredients, as previously described. This suggests that the in ovo feeding approach is an emerging in vivo method that can assess bioactive compounds with potential nutritional benefits. 

## Figures and Tables

**Table 1:** Studies of in ovo nutrients administration. BBM: brush border membrane.

Injected Substances	Aims	Injected Target	Infection Time	References
Extract of Laminaria species of seaweed	development of duodenum	air chamber	Day 12	[37]
Raffinose and stachyose	iron bioavailability, BBM functionality, gut microflora population	amniotic fluid	Day 17	[41]
Extract of chickpea and lentil, duck egg white peptides	calcium bioavailability, BBM functionality, gut microflora population	amniotic fluid	Day 17	[42]
Extract of beta-glucans, Transgalactooligosaccharides	hatchability, gut microflora population	air chamber	Day 12	[43]
Extract containing laminarin and fucoidan; Transgalactooligosaccharides from milk lactose	muscle, lipid oxidation of meat	air chamber	Day 12	[44]
Inulin, Galactooligosaccharides (GOS), *Lactococcus lactis*	transcriptomic prolife of spleen, cecal tonsils, and large intestine	air chamber	Day 12	[45]
Raffinose	gut health and immune system	air chamber	Day 12	[46]
*Silybum marianum* extract	immune system	amniotic fluid	Day 17	[47]
Inulin, *Enterococcus faecium*	BBM functionality, gut microflora population, short-chain fatty acid content	amniotic fluid	Day 17	[48]
Inulin, transgalactooligosaccharides, *Lactococcus lactis*	gut health and short-chain fatty acid content	air chamber	Day 12	[49]
Inulin, *Lactococcus lactis*	immune-related gene expression	air chamber	Day 12	[50]
Inulin, transgalactooligosaccharides, *Lactococcus lactis*	digestive potency of pancreas	air chamber	Day 12	[51]
Wheat prebiotics	iron bioavailability, gut microflora population	amniotic fluid	Day 17	[52]
Daidzein	BBM functionality, gut microflora population	amniotic fluid	Day 17	[53]
Inulin	iron bioavailability, gut functionality	amniotic fluid	Day 17	[54]
Raffinose, *Lactococcus lactis*	muscle fiber	air chamber	Day 12	[55]
Mannan oligosaccharides	small intestine development	amniotic fluid	Day 17	[56]
Dextrin, maltose, sucrose	mucin gene expression	amniotic fluid	Day 17	[57]
Zinc-methionine	zinc status, small intestine development	amniotic fluid	Day 17	[58]
β-hydroxy-β-methyl butyrate, Dextrin, maltose, sucrose	small intestine development	amniotic fluid	Day 17	[35]

**Table 2:** Functional gene expression and immune system response in the in ovo prebiotic administration model.

Gene	References
**Section A: Functional Gene Expression**	
Aminopeptidase (AP)/leucine aminopeptidase (LAP)	[41,42,53,56,58,122]
Sucrose isomaltase (SI)	[35,41,42,53,56,58,122]
Sodium glucose transporter 1 (SGLT1)	[41,53,56,58]
ATPase	[53,58]
Peptide transporter 1 (PepT1)	[56]
**Section B: Immune system**	
CD3, CD45, CD56, chB6	[46]
CD80	[50]
TLR2, TLR4	[46,56]
**Section C: Cytokine**	
IL-1β, IL-10	[46]
IL-4, IL-6, IL-8, IL-18, IL-12P40	[50]
IFN-β, IFN-γ	[50]
